# Ultra-Wide Band and Multifunctional Polarization Converter Based on Dielectric Metamaterial

**DOI:** 10.3390/ma12233857

**Published:** 2019-11-22

**Authors:** Ju Gao, Yiming Zhang, Yang Sun, Qiang Wu

**Affiliations:** Faculty of Information Technology, Beijing University of Technology, Beijing 100124, China; gaoju@bjut.edu.cn (J.G.); ymzhang@bjut.edu.cn (Y.Z.); sunyang@bjut.edu.cn (Y.S.)

**Keywords:** polarization conversion, metamaterial, ultra-wide band, high efficiency

## Abstract

Polarization has always been an important issue in modern communication systems, especially in sensitive measurements. Conventional polarization converters show limited applications due to their large size and narrow bandwidth. In this paper, we demonstrate an ultra-wide band, multifunctional, and highly efficient metamaterial-based polarization converter that is capable of converting a linearly polarized wave into its cross-polarized wave and circularly polarized wave over different frequency bands. The design principle is based on the field transformation theory and the anisotropic plate is made with high/low permittivity strip metamaterials. The simulation results show that the metamaterial-based polarization converter is able to achieve linear-to-linear conversion over 11.5–12.6 GHz, and linear-to-circular conversion over two frequency bands, 3.0–11.5 GHz and 12.6–17.0 GHz, with an average polarization conversion efficiency over 90%. The polarization converter proposed in this paper provides an important stepping stone for future communication systems’ polarization control and can also be extended to higher frequency bands.

## 1. Introduction

Electromagnetic waves play an important role in the field of modern communication engineering, and people are increasingly hoping to achieve perfect control of electromagnetic waves with devices such as polarization converters [1,2,3,4,5], lenses [6,7], and phase modulators [8,9,10]. The amplitude and phase control are greatly developed in devices such as amplifiers and power dividers, and metasurfaces [11,12,13,14,15]. The regulation of polarization, however, is still insufficient and can be further developed. Future communication systems will be based on multiple polarization devices to exchange information, so polarization control is essential. Compared with traditional polarization control devices based on dichroic crystals and birefringent materials, metamaterials present exotic properties, such as a lighter, smaller, and simpler design [16,17]. There are several polarization converters based on metamaterials that show good performance from microwave bands to optics. Cui et al. showed a double V-shaped metamaterial as a reflective polarization converter [18] and Gansel et al. presented a broadband circular polarizer in the infrared band [19]. Chiral property is one of the essential characteristics in metamaterial polarization converter design. Wu et al. developed a new method based on toroidal dipoles to construct a polarization converter [20]. Hua et al. verified the use of micron-scale graphene to achieve linearly polarized conversions [21]. Yang et al. combined high refractive index silicon crystals and a silver ground plane to form a high-efficiency optical band polarization converter [22]. Compared to reflective converters, it is very difficult to construct an ultra-broadband-transmitted polarization converter [23,24]. The aforementioned polarization converter designs, however, cannot outperform ultra-thin, broadband, and multifunctional converters, which will be of great demand in future applications.

The above problems can be better solved by using all-dielectric metamaterials. In this paper, the field transformation theory is used to construct the constitutive parameter matrix required for polarization conversion. By solving the parameter matrix, the coupling effect between the structural units is used to approximate the diagonal tensor in the parameter matrix, and finally achieve polarization conversion in the microwave region over an ultra-wide band through a dielectric strip metamaterial. The dielectric strip metamaterial is capable of transforming the incident linearly polarized wave into its cross-polarized wave as well as circularly polarized wave from 3 GHz to 17 GHz, with a main polarization conversion ratio of 90%. Compared to other ultra-thin or broadband designs, the polarization converter proposed in this paper shows better performance in terms of device volume and operating bands, which makes it more convenient in communication system integration than other devices.

## 2. Materials, Methods, Design, Results, and Discussion

The most critical objective here was to achieve a different polarization status on both the sides of a medium. Firstly, the field transformation matrix should be set-up based on the polarization conversion requirement. Secondly, the required constitutive parameter equation matrix must be solved according to the field transformation theory and boundary conditions. Finally, an anisotropic dielectric metamaterial must be used to achieve the solution of the constitutive parameter matrix. Based on frequency-independent research methods, the dielectric metamaterial constructed here can convert the incoming wave in the required space over an ultra-wide band.

Based on the field transformation theory [25], the relationship between the transformed field and the original field is as follows:

(1) $$\left[\begin{array}{l} E_{z}\\ iH_{z} \end{array}\right]\;=\;\left[\begin{array}{cc} \cos\varphi & -\sin\varphi \\ \sin\varphi & \cos\varphi \end{array}\right]\left[\begin{array}{l} E_{z}^{\left(0\right)}\\ iH_{z}^{\left(0\right)} \end{array}\right],$$

where *φ*(*x*,*y*) is a function related to the constitutive parameter, which determines the relative permittivity and permeability distribution in a specified region based on field transformation. The permittivity and permeability can be described as:

(2) $$\varepsilon \;=\;\left[\begin{array}{ccc} n & 0 & A_{y}\\ 0 & n & -A_{x}\\ A_{y} & -A_{x} & n \end{array}\right],\mu \;=\;\left[\begin{array}{ccc} n & 0 & -A_{y}\\ 0 & n & A_{x}\\ -A_{y} & A_{x} & n \end{array}\right],$$

where $A_{x}\;=\;(1/k_{0})\partial \varphi /\partial x$ and $A_{y}\;=\;(1/k_{0})\partial \varphi /\partial y$ determine the coupling effect in the transformation medium, *n* is the refractive index of the isotropic plate. As the constitutive parameter matrix based on field transformation was diagonally symmetric, the coupling relationship between TE polarization (along *x* axis) and TM polarization (along *y* axis) was enhanced. Hence, the polarization status will be different when the incident wave passes through the medium with the above-mentioned constitutive parameter distributions. After obtaining the correspondence between the virtual space and the real space by field transformation, the impedance in the required region can be perfectly controlled. If the electric field distribution and the magnetic field distribution in the specific region were rearranged based on the required electromagnetic characteristics, this transformation method would ensure that the reflection at the theoretical level was not excessive due to the impedance mismatch.

Then, by constructing a similar dispersion surface, all magnetic responses were included in the dielectric permittivity. This idea was similar to the so-called parameter simplification idea in optical transformation, so as to simplify the design and facilitate processing [26]. The permittivity and permeability here can be written as:

(3) $$\varepsilon \;=\;\left[\begin{array}{ccc} n^{2} & 0 & 2A_{y}\\ 0 & n^{2} & -2A_{x}\\ 2A_{y} & -2A_{x} & n^{2} \end{array}\right],\mu \;=\;1.$$

**Figure 1:**
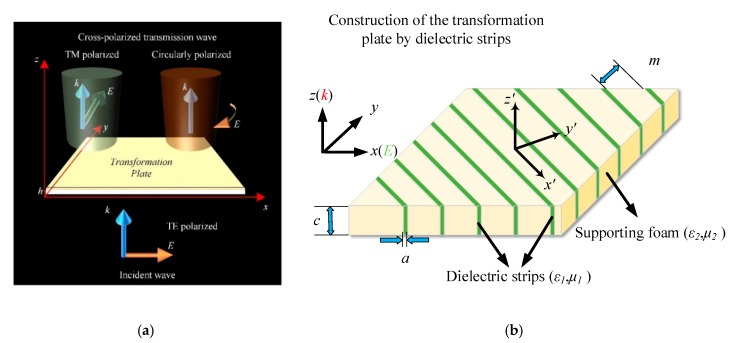
(**a**) Schematic of the multifunctional polarization converter. (**b**) Construction of the polarization converter.

Assuming that the constitutive parameters of the conversion medium required in a particular region are linear and the transformation plate thickness is *h*, *φ* can be expressed as *φ* = *φ*_max_*y/h*, *φ*_max_ = π/4, and *φ*_max_ = π/2 and the process of y = 0 and y = h/2 corresponds to the conventional quarter-wave plate and half-wave plate, respectively. Therefore, it can be calculated that *A*_x_ = 0 and *A*_y_ = *φ*_max_/(*k*_0_*h*). The required polarization conversion structure is shown in Figure 1.

Figure 1a shows the schematic of what we want from the field transformation plate. Figure 1b shows the construction method of the transformation plate by using dielectric strips. To achieve this particular constitutive parameter, an anisotropic material needs to be built in the specific space. According to the effective medium theory [27,28], it can also be obtained by using a set of strips with different permittivity and placing the strips at an angle of 45 degrees with the incident electric field vector. The construction of the polarization converter is shown in Figure 1b. The transformation coordinate x′y′ differed from the original xy coordinate by 45 degrees. Based on the theory in [25,26,27,28], the effective permittivity along each direction *ε*_x*,*y,z_ equaled the permittivity matrix in Equation (3). As the dielectric strips were located 45 degrees different from the original coordinate system, *ε_x,y,z_* in reference [27] was transformed to *ε_x′,_*_y*′,*z*′*_ in our design. Assuming that the relative dielectric permittivity of the two different strips are *ε*_1_ and *ε*_2_, then the main diagonal of the permittivity matrix equals *ε_x′_*, $\frac{1}{\varepsilon_{x^{\prime }}}=\frac{1}{1+\eta }(\frac{1}{\varepsilon_{1}}+\frac{\eta }{\varepsilon_{2}})=\frac{1}{n^{2}}$ and the sum of the other diagonals equals *ε*_y*′*_, $\varepsilon_{y^{\prime }}=\frac{\varepsilon_{1}+\eta \varepsilon_{2}}{1+\eta }=\left|2A_{y}\right|+\left|2A_{x}\right|$ [26], where *ε*_1_ and *a* are the dielectric permittivity and width of the high dielectric permittivity strip, respectively, and *ε*_2_ and *m* are the dielectric permittivity and width of the low dielectric permittivity strip, respectively. *ƞ* is the width ratio of two dielectric strips. *ε*_x*,*y*,*z_ refers to the effective permittivity in the *x*, *y*, or *z* direction. The above formula can be obtained by introducing Equation (3), *n* = 1.25, *A*_x_ = 0, and *A*_y_ = 1.8. It can be calculated that when the low dielectric permittivity strip was air, the required dielectric permittivity of the high dielectric permittivity strip was about 50 and the width ratio of the two was about 17. The polarization conversion of the incident wave was achieved by utilizing the coupling between the strips with different dielectric permittivities.

It can be seen from the above derivation that the required constitutive parameter matrix of the polarization conversion was distributed in a symmetrical form. Hence, we proposed to achieve it by rotating an artificially designed material slab with fine dielectric gratings by 45 degrees in the *xoy* plane. The proposed structure of the polarization converter is shown in Figure 1b. The permittivity of the dielectric strips was 50 + 0.008i. The parameters of the dielectric plate were as follows: the thickness *h* of the plate was 2.45 mm, the width *a* was 0.33 mm, and the distance *m* between the plates was 5.8 mm. The yellow parts in Figure 1b indicate the supporting medium, which was vacuum in the simulation and foam in the fabrication. The thickness along the incident wave was about 1/10 wavelength.

One of the most important parameters in our work was the polarization conversion ratio (PCR), which is the ratio of the converted incident energy. As conversion occurs in the transmitted wave over the operating band, the PCR can be calculated as:

(4) $$PCR\;=\;\frac{P_{c}}{P_{i}}\;=\;\frac{P_{tc}}{P_{i}},$$

where *P_c_* is the converted energy and *P_i_* is the incident energy. *P_tc_* refers to the converted energies in the transmitted wave. As polarization conversion occurs in the transmittance component and the reflection in this instance is very small, the formula was simplified to the last expression.

The simulations were accomplished in the CST Microwave Studio 2018 with a frequency domain solver. Two floquet ports with different polarized modes were set on both sides of the polarization converter. The cross-polarized wave can be detected and received by the receiving port. Thus, S_2(cross),1(TE)_ was equal to the polarization conversion ratio, where the cross-polarized wave in port 2 can be a TM polarized linear wave, right-handed circularly polarized (RHCP) wave, or left-handed circularly polarized (LHCP) wave.

**Figure 2:**
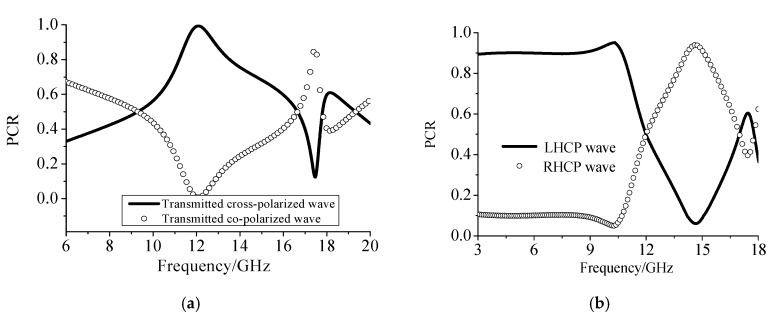
The transmitted PCR excited by a TE polarized wave. (**a**) Co-polarized (TE) and cross-polarized (TM) polarized component in the transmitted wave. (**b**) TE to circular polarization conversion ratio in the transmitted wave.

Figure 2 shows the scattering coefficients of different polarization components in the transmitted wave when the incident electromagnetic wave was a linearly polarized TE wave. It can be seen from Figure 2a that the designed structure had a good conversion efficiency. At the resonant frequency of 12.05 GHz, the component of the TM polarized wave reached a peak of 0.9998 in the transmitted wave, which meant that the TE polarized wave was 99.98% transformed into the cross-polarized TM wave after passing through the dielectric grating at this resonant frequency. That is, the polarization conversion efficiency of the dielectric strip at the resonant frequency was 99.98%. The structure operated as a half-wave plate over this frequency band, which meant that it can transform not only linearly polarized wave into its cross-polarized wave, but also into its circularly polarized wave. Compared to the other polarization converters published, the design in this paper showed good performance on conversion efficiency. The relative operation bandwidth was 10%.

Meanwhile, in Figure 2b, when the incident wave was a linearly polarized wave, the circularly polarized component in the transmitted wave occupied a large proportion. When the frequency was 3 GHz to 11.5 GHz, more than 90% of the linearly polarized (TE) wave converted into a left-handed circularly polarized wave in the transmitted wave. When the frequency was around 12 GHz, the incident linearly polarized wave was transformed into its cross-polarized wave, as shown in Figure 2a. When the frequency was higher, at 12.6 GHz to 17 GHz, the incident wave was converted into a right-handed circularly polarized wave in the transmitted wave. The half-wave plate relative bandwidth was 9.05% and the quarter-wave plate relative bandwidths were 117.24% and 28.19%.

## 3. Inherent Mechanism Explanation

The principle of polarization converter was also studied by equating the incident wave into two mutually perpendicular electromagnetic waves. Since the gratings were placed at an angle to the electric field of the incident electromagnetic wave, the electric field can be decomposed into two orthogonal electric fields: one was perpendicular to the dielectric strip and the other was parallel to the direction of the dielectric strip. By separately studying the excitation differences of the two orthogonal directions of the electric field to the dielectric strips and recombining them together, it can help to further understand and clarify the mechanism of polarization conversion.

**Figure 3:**
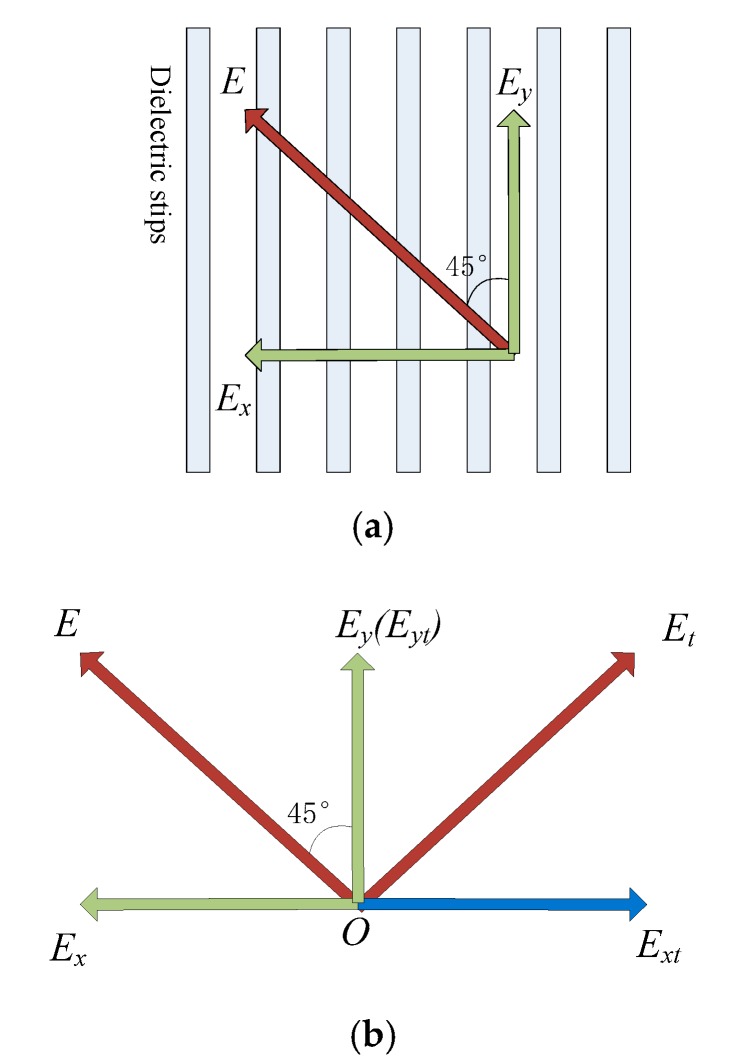
(**a**) Orthogonal decomposition of the incident wave. (**b**) Schematic of polarization conversion in the transmitted wave component.

The inherent mechanism of polarization conversion is explained in Figure 3a,b. As shown in Figure 3a, the incident wave was decoupled into two orthogonal electric field components. Since the electric field of the electromagnetic wave was also exactly 45 degrees from the dielectric strip, the electric field component artificially decomposed into two equal-sized components with directions exactly perpendicular and parallel to the direction of the dielectric strip. Therefore, solving and superposing the decomposed *E*_x_ and *E*_y_ results, acquired by the excitation, equaled the electromagnetic response when the electric field and the dielectric strip were at an angle of 45 degrees.

In Figure 3b, it was assumed that the decomposed field in which the phase change occurred was *E*_x_ and the field transmitted out was *E*_x*t*_. Since the phase changed by 180 degrees, the incident electric field changed from the direction of the green field component to the opposite direction of radiation, the direction of the blue field component. As there was no phase change in the *E*_y_ field component, the transmitted wave in green color was consistent with the direction of the incident field, as shown in the figure, so the direction of the transmitted electric field changed from the radiation direction of *E* to *E_t_*. It can be seen from the figure that their radiation directions were orthogonal to 90 degrees, thereby achieving linear polarization conversion in the transmitted wave.

**Figure 4:**
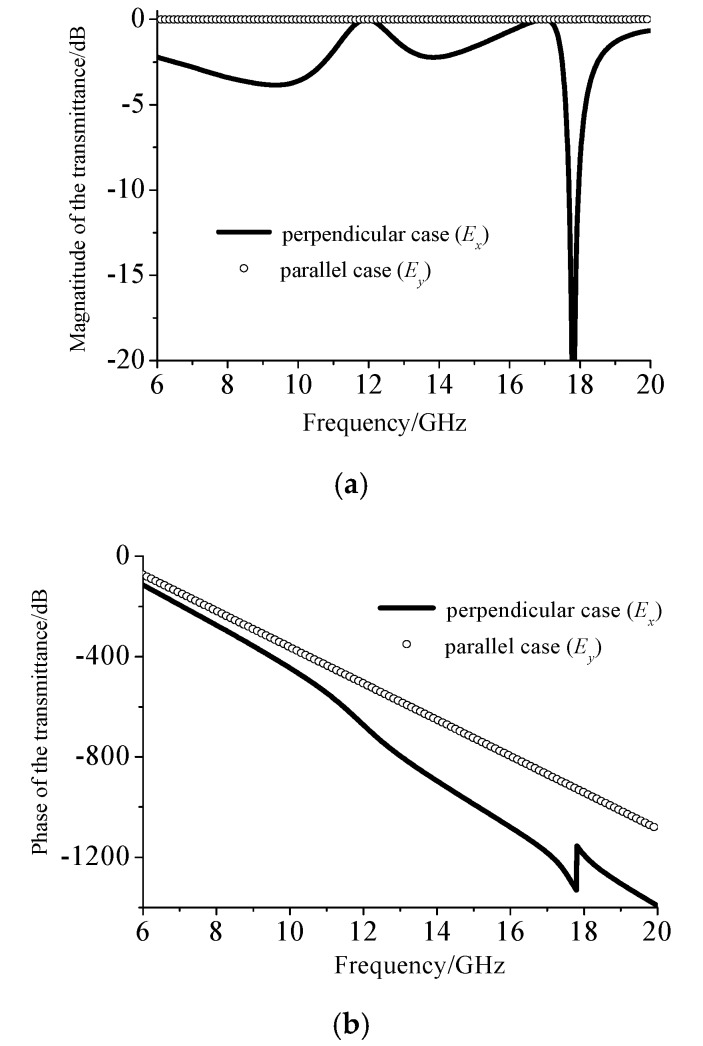
Simulated results of the model in Figure 3a. (**a**) The amplitude in the transmitted wave excited by two orthogonal decomposed waves. (**b**) The phase in the transmitted wave excited by two orthogonal decomposed waves.

Figure 4 shows the response of the dielectric plate when excited by two orthogonal electric fields *E*_x_ and *E*_y_ (with respect to Figure 3). Figure 4a shows the magnitude of the electromagnetic response excited by the dielectric strips when the electric field was perpendicular or parallel to the dielectric strips. It can be seen that when the dielectric strip was excited by an electric field parallel or perpendicular to them, their scattering field radiation intensity remained substantially the same over 12 GHz.

Figure 4 shows the simulated results of the model from Figure 3 to explain why the proposed metamaterial can realize linear-to-linear polarization conversion. The only operation band that was of concern is around 12 GHz (corresponding to Figure 2a). Figure 4a is a diagram showing that the electric field components of two orthogonal decompositions at 12 GHz can excite an equal-amplitude excitation field. The reason that the proposed metamaterial can realize linear-to-circular polarization conversion was based on the field transformation theory. The calculated phase accumulation φ_max_ was π/4, which meant that the polarization converter was essentially a quarter-wave plate.

Figure 4b shows the phase of the electromagnetic response field excited by the dielectric strips when the incident electric field was perpendicular or parallel to the direction of the dielectric strips. The electromagnetic response of the two orthogonally excited electric fields after the illumination of the dielectric strips was always different for the phase case, but the phase changes of both cases were linear and there was no phase shifting.

**Figure 5:**
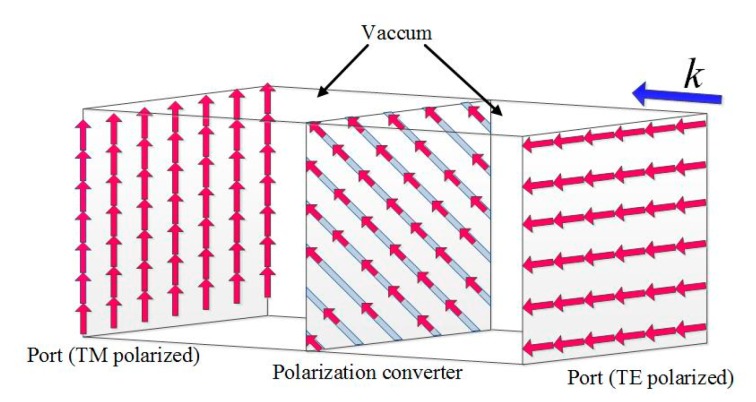
The schematic of linear polarization conversion around 12 GHz.

The field distribution of the polarization conversion process is also illustrated in Figure 5. While the incident wave transmitted through the dielectric gratings, electromagnetic waves would experience faster phase accumulation inside due to high permittivity strips without large substrate thickness. The incident linearly polarized wave would be transformed into its cross-polarized wave around 12 GHz in the transmittance wave.

The polarization conversion mechanism of the proposed metamaterial was based on the fact that one orthogonal component of the decomposed incident electric field had faster phase progression within the metamaterial compared to the other component, which resulted in the quarter-wave plate. The reason why it behaved as a half-wave plate over 12 GHz was that the phase progression changed with frequency and the left circularly polarized wave component amplitude equaled the right circularly polarized wave. Two circularly polarized waves combined to form a linearly polarized wave and the proposed metamaterials behaved as a half-wave plate over 12 GHz.

A comparison between our work and the published broadband polarization converters is shown in Table 1. It can be seen from the table that the multifunction polarization converter constructed according to the field transformation theory has obvious advantages in terms of relative bandwidth. This also means that there is a clear advantage in the comprehensive regulation of electromagnetic waves. It can be seen from the comparison results that the polarization converter of this paper can achieve polarization conversion over the 3.0–11.5 GHz band, and the relative working bandwidth was 117%. The relative working bandwidth of a converter, from one of the previous studies, in the microwave band with broadband polarization conversion as the bright spot was over 80% in the reflective wave, but much less in the transmitted wave (less than 10%). The PCR in the reflection system was higher because of the metal ground on the back to decrease the energy loss. Therefore, the polarization converter based on the field transformation theory has great advantages in relative working bandwidth.

The broadband conversion performance can be explained through the field transformation theory. The theory starts from the field distribution requirements on both sides of the interface, and sets up the limited space matrix for constitutive parameters. While in the process of field transformation, each step of the transformation was frequency-independent. Therefore, the polarization converter obtained by this frequency-independent theory has excellent broadband performance.

**Figure 6:**
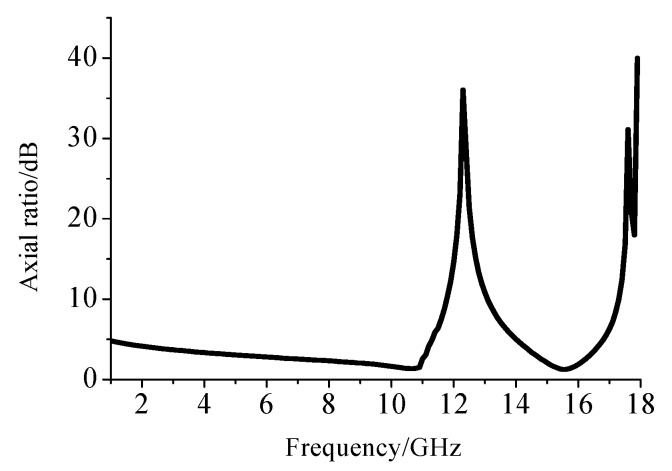
Axial ratio of the polarization converter over the operating band.

In order to explain the conversion ability of the circularly polarized wave when the structure was excited by a linearly polarized wave, the axial ratio characteristic in the transmitted wave was analyzed. The axial ratio is the ratio of orthogonal components of an E-field. A circularly polarized field is made up of two orthogonal E-field components of equal amplitude and 90 degrees out of phase. In engineering, we regard one wave as circularly polarized when its axial ratio is less than 3 dB and linearly polarized when its axial ratio is larger than 15 dB. Figure 6 shows the axial ratio of the proposed polarization conversion structure over the entire operating frequency band.

Figure 6 shows the axial ratio of the circularly polarized component of the transmitted wave over the entire frequency band with a determined transmission angle. It can be seen from the figure that when the frequency reached 3 GHz, the axial ratio of the structure was less than 3 dB, which indicated that the components in the transmitted wave were circularly polarized at this frequency. When the incident wave frequency was between 3–11 GHz, the circularly polarized wave was dominant in the transmitted wave component. When the frequency was increased to around 12 GHz, the axial ratio reached a maximum of 38 dB, which meant that all of the transmitted wave components had been converted into linearly polarized waves at that time. When the frequency continued to rise, the axial ratio fell again. When the frequency reached 15.3 GHz, the circular polarization axis ratio was 1.2 dB. The incident wave at this time was twisted into a circularly polarized wave in the transmitted wave.

It can be seen that the polarization converter proposed in this paper can achieve polarization conversion over 3 GHz to 17 GHz and multifunctional polarization conversion according to the frequency band without changing the structure of the device and other additional conditions.

**Figure 7:**
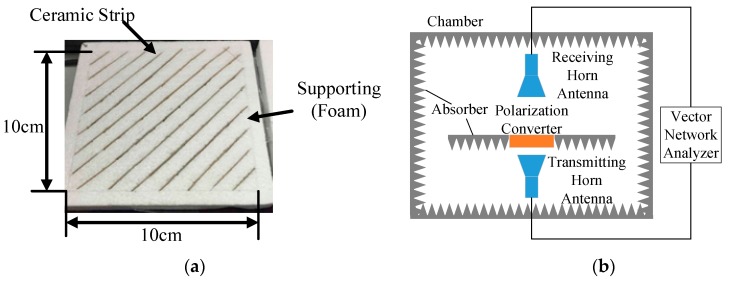
Experiment set-up of the polarization converter. (**a**) Fabricated polarization converter sample. (**b**) Experiment polarization converter.

This polarization converter was also fabricated and measured. The experiment results showed good agreement with the calculated and simulated results. The schematic of the experiment and sample is shown in Figure 7.

**Figure 8:**
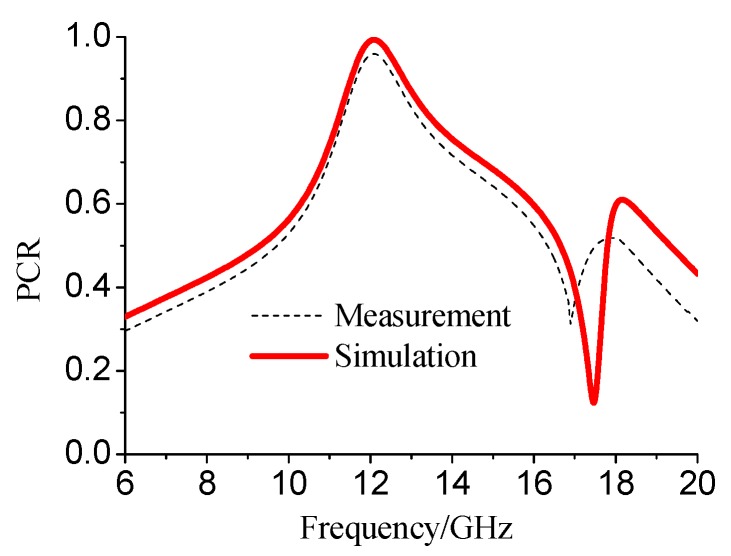
Experimental and simulated polarization conversion results.

The sample was fabricated by ceramic technology and the composite inside was barium titanate trioxide (BaTiO_3_) polymer powder. BaTiO_3_ powder is a ferro-electric compound material with high permittivity and low dielectric loss. It is one of the most widely used materials in electronic ceramics and exhibits excellent dielectric properties in the microwave region. The polarization converter was measured in an antenna chamber with absorbers around. The whole experiment process revolved around linearly polarized conversion due to the experimental conditions. The agreement in results for simulation and experiment over 11.5 GHz to 12.6 GHz was convincing to verify the theory and simulation. The transmitting and receiving horn antennas were placed parallel and vertically polarized to the ground, respectively. The experiment results are shown in Figure 8.

As shown in Figure 8, the measured results had good concordance with the simulated results as there was no resonant frequency shift or obvious decrease in PCR. The best conversion performance appeared around 12.08 GHz and the ratio was 95.9%. The relative linear polarization conversion bandwidth was 8.6%. The little differences between the simulation and experiment results were in the operation bands, which were caused by the loss in the ceramic strip and foam. Thus, the measured results support the conversion theory effectively.

## 4. Conclusions

In view of the problem that the electromagnetic structure cannot independently respond to the electric and magnetic fields of the incident wave transverse component, we used an all-dielectric structure to construct a multifunctional polarization converter with high efficiency and ultra-broadband. The structure utilized the mutual heterogeneity of the constitutive parameter matrix to enhance the ability of transverse electromagnetic field regulation. Based on the field transformation theory, the required dielectric permittivity and permeability distribution law were deduced. The high-efficiency multifunction wide-band polarization converter was designed based on the field transformation theory and by using the coupling effect of the high-low dielectric permittivity strips. Simulation results showed that the proposed structure can realize the whole polarization state conversion. The polarization conversion efficiency was as high as 99.98%. The half-wave plate relative bandwidth was 9.05% and the quarter-wave plate relative bandwidths were 117.24% and 28.19%.

## Figures and Tables

**Table 1:** Comparison with other published wide band polarization converters.

Works	Operation Mode	Efficiency (Measurement)	Absolute Bandwidth	Relative Bandwidth
Our work	Transmitted both quarter- and half-wave plates	95.9%	3.0–11.5 (GHz)	117%
[1]	Reflective half-wave plate	93%	6.67–17.1 (GHz)	87.7%
[2]	Reflective both quarter- and half-wave plates	90%	3.9–7.9 (GHz)	66.8%
[4]	Reflective half-wave plate	90%	17.3–42.2 (GHz)	87.47%
[18]	Reflective half-wave plate	80%	12.4–27.96 (GHz)	77%
[23]	Transmitted half-wave plate	27.5%	4.68–4.92 (GHz)	5%
[24]	Transmitted quarter-wave plate	90%	2.2–2.6 (GHz)	8%
[29]	Reflective half-wave plate	98.4%	14–16.5 (GHz)	16%
